# The Attentional Capture Debate: When Can We Avoid Salient Distractors and When Not?

**DOI:** 10.5334/joc.251

**Published:** 2023-07-06

**Authors:** Jan Theeuwes

**Affiliations:** 1 Cognitive Psychology, Vrije Universiteit Amsterdam, Netherlands; 2 Institute Brain and Behavior Amsterdam (iBBA), Netherlands; 3 William James Center for Research, ISPA-Instituto Universitario, 1149-041, Lisbon, Portugal

**Keywords:** Visual search, Learning, Attention

## Abstract

There has been a long-standing debate concerning whether we are able to resist attention capture by salient distractors. The so-called “signal suppression hypothesis” of Gaspelin and Luck (2018) claimed to have resolved this debate. According to this view, salient stimuli “naturally attempt to capture attention”, yet attention capture may be prevented by a top-down inhibitory mechanism. The current paper describes the conditions in which attention capture by salient distractors can be avoided. Capture by salient items can be avoided when the target is non-salient and therefore difficult to find. Because fine discrimination is needed, a small attentional window is adapted resulting in serial (or partly serial) search. Salient signals outside the focused attentional window do not capture attention anymore not because they are suppressed but because they are ignored. We argue that in studies that have provided evidence for signal suppression, search was likely serial or at least partly serial. When the target is salient, search will be conducted in parallel, and in those cases the salient singleton cannot be ignored nor suppressed but instead will capture attention. We argue that the “signal suppression” account (Gaspelin & Luck, 2018) that seeks to explain resistance to attentional capture has many parallels to classic visual search models such as the “feature integration theory” (Treisman & Gelade, 1980), “feature inhibition” account (Treisman & Sato, 1990), and “guided search” (Wolfe et al, 1989); all models that explain how the serial deployment of attention is guided by the output of earlier parallel processes.

## Introduction

Over the last 30 years, there has been a fierce debate regarding the extent to which visual selection is controlled by us or controlled by the outside world. In other words: Do we attend the flashing lights of a police car because we want to attend these lights or are these lights so salient that they pull attention automatically? The debate has been about the extent to which visual selection is controlled in a voluntary, top–down way or in an automatic, bottom–up way (e.g., see reviews Burnham, 2007; Corbetta & Shulman, 2002; Theeuwes, 2010, 2019; Luck, Gaspelin, Folk, Remington, & Theeuwes, 2021). If at all time, attention is allocated voluntarily in line with the behavioral goals, selection can be considered to be top-down and voluntary (Folk, Remington, & Johnston, 1992). However, if objects are salient, often times these objects are selected even when observers try to ignore them. In these cases, selection is considered to be bottom-up and automatic (Theeuwes, 2010, 2018). One speaks of attention capture (Theeuwes, 1992) when the object captures attention and oculomotor capture (Theeuwes et al., 1998) when such a salient object not only captures attention but also triggers an exogenous saccade to the location of the event.

In a recent TICS paper Gaspelin and Luck (2018a) claimed to have provided a solution for the longstanding debate. They argued that salient stimuli indeed “naturally attempt to capture attention”; yet they also argued that “attention capture can be prevented by a top-down inhibitory mechanism”. This solution elegantly incorporates both sides of the attention capture debate. If the salient stimulus is not suppressed, it will capture attention consistent with stimulus-driven accounts; however, if top-down control is exerted, the salience signal is suppressed so that it no longer captures attention. This idea is supported by several empirical findings including studies showing below baseline suppression of a salient singleton when observers engage in what is called the feature search mode (Bacon & Egeth, 1994). When this search mode is used, observers search for a specific feature and only in these circumstances, they are able to impose top-down control allowing sub-baseline suppression of salient distractors. This has led to the signal suppression hypothesis (Gaspelin & Luck, 2018a) which seems to provide a new view on the attention capture debate. In a recent review, Luck et al. (2021) described the progress made in the attention capture debate with a prominent role for the signal suppression hypothesis.

Recently, the signal suppression hypothesis has received a lot of support from various studies that all seem to embrace the idea that a salient signal can be suppressed without attending it (Chang & Egeth, 2019, 2021; Gaspelin et al., 2015; Vatterott et al., 2018; Won et al., 2019; Won & Geng, 2020). There is also evidence from studies using eye movements (Gaspelin et al., 2017, 2019; Gaspelin & Luck, 2018b), event-related potentials (Feldmann-Wüstefeld et al., 2020; Gaspar & McDonald, 2014; Gaspelin & Luck, 2018a, b, c; Sawaki & Luck, 2010; Weaver et al., 2017), and single-unit recordings in monkeys (Cosman et al., 2018).

It is clear that the *signal suppression hypothesis* is a major step forward in the attentional capture debate (see for a review Luck et al., 2021). Yet the boundary conditions when capture by salient distractors can be avoided and when not, are blurry and it is crucial for the current debate that these boundary conditions are clearly demarcated. The current paper sets these boundary conditions by explaining when a signal of a salient distractor can be avoided and when a salient signal inevitably is selected.

## Signal suppression vs. the feature inhibition account

Dating back to the classic work of Neisser (1967) and Treisman and Gelade (1980), it is known that visual search can be conducted either in parallel across the visual field or serially one by one (or serially in clumps). According to the feature integration theory (FIT) of Treisman and Gelade (1980), when the target has distinguishing features (such as color, orientation, or size), its presence can be registered across the visual field by means of preattentive, parallel processing. If, however, the target is more complex or needs binding of its basic features, it requires a serial shifting of the focus of attention from one (clump of) objects to the next until the target is found or all objects have been inspected. Over the years, FIT has been revised to include the possibility that feature information can guide the (serial) deployment of attention (Wolfe et al., 1989; Wolfe 1994, 2021; Treisman & Sato, 1990). For example, in displays consisting of red and green items, when observers know that the target is red, they will search serially through the red items only, while avoiding search among the green items (Egeth, Virzi, & Garbart, 1984; Zohary & Hochstein 1989). Specifically, Kaptein, Theeuwes, and Van der Heijden (1995) showed that this top-down guided search can be implemented flexibly, as participants were able to selectively select the cued color and ignore the uncued color on a trial-by-trial basis without any performance decrement.

The message from these studies is that when search is serial, it can be guided by top-down knowledge in a highly flexible way (Wolfe, 2021). To account for findings like these, Treisman and Sato (1990) put forward the “feature inhibition account”, arguing that the master map of locations may receive inhibition from feature maps containing task-irrelevant features, thereby reducing the interference from distractors. While the original signal suppression account of Sawaki and Luck (2010) that assumed that any salient (“attend-to-me”) distractor can be suppressed, was notably different from the feature inhibition account of Treisman and Sato (1990), the updated version of Gaspelin and Luck (2018a) has many parallels to the original feature inhibition account. Indeed, according to the updated signal suppression account, suppression does not work on saliency per se but instead only operates on specific features (i.e., a specific color) and observers must ‘know’ the color they need to avoid. In essence, the new signal suppression account of Luck and colleagues may not be that different from the feature inhibition account of Treisman and Sato (1990).

Yet, what makes these notions different is that the classic visual search models such as “feature inhibition” (Treisman & Sato, 1990), and “guided search” (Wolfe et al, 1989) seek to explain the guidance of the (serial) deployment of attentional resources throughout the display while the Gaspelin and Luck’s feature suppression account is used to explain why in some conditions, salient singletons do not capture attention. Nevertheless, the notion of the signal suppression account that “*attention capture can be prevented by a top-down inhibitory mechanism*” is quite similar to features that are inhibited according Treisman and Sato’s feature inhibition account. As argued below, the conditions in which the signal-suppression account explains the absence of attentional capture all invoke serial or partly serial search and therefore the absence of attentional capture by salient distractors can also be explained by the classic visual search models of Treisman and Gelade (1980) and Wolfe (1994).

## When can attentional capture be avoided?

**Figure 1:**
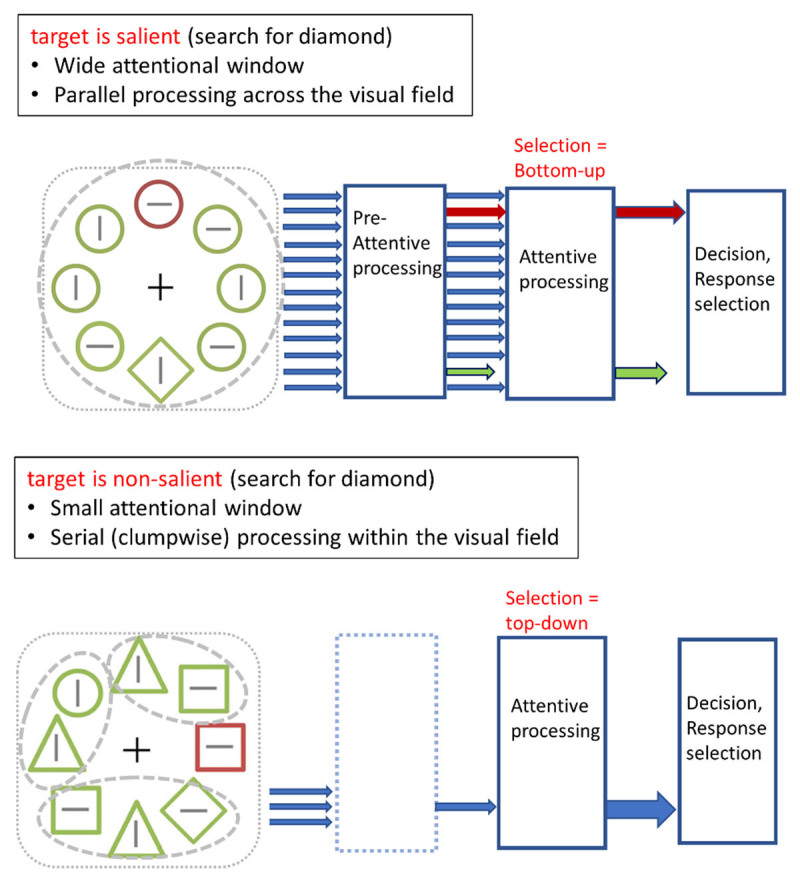
Illustration of search for a shape singleton target (diamond) when the target is salient (top of figure) and when the target is non-salient (bottom of Figure). When the target is salient, the attentional window is wide encompassing all items in the display. Because the target is salient it can be found by preattentive parallel search. During preattentive parallel search there is no top-down control and therefore the most salient singleton (the color singleton) captures attention. When the target is non-salient (bottom of Figure), the attentional window is reduced to be able to find the non-salient target. Because search proceeds serially in clumps, the color singleton no longer captures attention. Dependent on the difficulty of search, the size of clumps that are searched can vary while search proceeds (e.g., homogenous clumps of items can be discarded in large groups). Because the salient singleton no longer captures attention, selection is considered to be under top-down control.

Figure 1 illustrates the conditions when there is attention capture by a salient singleton distractor and conditions when attention capture can be avoided. In this example illustrated in Figure 1, participants search for a diamond. If the diamond is salient (top of the figure), participants spread attention across the visual display such that the attentional window encompasses all items in the display. In these circumstances, initially search proceeds in parallel across all items in the display. If, under those circumstances, the distractor singleton is more salient than the target singleton, there will be attentional capture by the distractor; representing the exact conditions that were tested in the original Theeuwes (1992) paper. If, however, the very same diamond target is surrounded by heterogenous nontarget elements (bottom of the figure), it becomes relatively non-salient forcing some serial-like clump-wise search. Dependent on the difficulty of search (i.e., how “non-salient” the target is in any given display), the size of the attentional window may vary from very focused to rather wide during a particular search episode (e.g., homogenous clumps of items can be discarded in large groups). During serial search, the salient distractor outside the attentional window is simply ignored and therefore no longer captures attention.

Note that for capture to occur (top of the figure) not only the target needs to be salient, but also the distractor singleton needs salient and more salient than the target singleton. For example, Theeuwes (1992) showed that when a color distractor singleton is less salient than the target singleton, the distractor is ignored, not because of top-down control but simply because if attention is divided across the visual field (top of Figure 1), the most salient element in the display captures attention regardless of whether it is the target or the distractor.

According to this analysis, attentional capture is avoided when the target is non-salient. In those conditions, search proceeds serially in clumps, the size of which is determined by signal-to-noise ratio to locate the target within a clump (e.g., the assumed attentional window; see Theeuwes, 1994, 2010). The assumed processes as described in Figure 1 are consistent with the findings of studies showing no attentional capture by a salient singleton distractor. Indeed, experiments that have shown that capture by a salient singleton can be avoided (Gaspelin et al., 2015; Chang & Egeth, 2019, 2021; Ma & Abrams, 2022; Wang & Theeuwes, 2020; Theeuwes, 2004; see Figure 4) all have used either heterogenous displays (as an example, Figure 1 bottom) or displays in which only a few items were presented (e.g., display sizes 4, 5 and 6). These findings have been interpreted as evidence that top-down control can prevent attentional capture.

## Small set sizes and/or heterogeneous displays

The critical question is then whether the signal suppression hypothesis can be applied to conditions in which both the target and the distractor are salient (top of Figure 1). If the signal suppression hypothesis has anything to say about the attention capture debate, it should show suppression of the irrelevant singleton distractor under exactly these circumstances. Even though there is a lot of empirical support the signal suppression account (e.g., Chang & Egeth, 2019, 2021; Gaspelin et al., 2015; Gaspelin et al., 2017, 2019; Gaspelin & Luck, 2018b, 2018c; Sawaki & Luck, 2010; Stilwell & Gaspelin, 2021), the account was never tested in conditions in which both the target and distractor singleton were salient (top of Figure 1). Indeed, in all of the above-mentioned studies the target was non-salient because either relatively small set sizes (4 or 6 elements) were used and/or because displays consisted of heterogeneous elements (involving shapes such as squares, hexagons, triangles, pentagons, circles; see Figure 1 bottom for an example). These very conditions render the target singleton as non-salient. According to this analysis, signal suppression only works in conditions in which the discriminability of the target is relatively low (see for example Figure 1 and 4, the diamond is hard to find among other elements).

One of most critical study that arrived at this conclusion was a recent study of Wang and Theeuwes (2020). They first replicated the original Gaspelin et al. (2015) findings that were critical for the signal suppression hypothesis. In Gaspelin et al. (2015), the additional singleton task (Theeuwes, 1991, 1992) was combined with a letter probe task. In 70% of trials, observers searched for a target shape while ignoring a color singleton. In 30% of trials, letter probes were briefly presented inside the search elements, and observers needed to report as many letters as possible. In conditions in which observers used the so-called singleton detection mode (cf. Bacon & Egeth, 1994), it was shown that observers reported more letters when these were presented at the location of the singleton distractor than presented at other nonsingleton distractors locations. This was considered as evidence for attentional capture by the salient distractor. Critically, however, when observers had to engage in the feature search mode, the accuracy for the letter at the irrelevant singleton distractor was reduced below the accuracy observed for letters at the nonsingleton distractors. Gaspelin et al. considered this sub-baseline suppression as decisive evidence for active suppression of the singleton-distractor location. The most critical experiment of Gaspelin et al. (2015) was their Experiment 4 in which the letters and search display were presented simultaneously. This version of their paradigm was used by Wang and Theeuwes (2020).

Wang and Theeuwes (2020) replicated the findings of Gaspelin et al. (2015) and showed that when engaged in feature search there is sub-baseline suppression of the distractor singleton. Yet, critically this was only found for small heterogeneous displays consisting of just 4 elements. For large display sizes (e.g., set size 10) there was no evidence for feature-inhibition but instead (and opposite to the predictions of the signal suppression account) there was evidence that the salient distractor captured attention. Indeed, instead of identifying fewer letters from inside the salient distractor (which would indicate suppression), observers correctly identified more often letters that were placed within the salient distractor (indicating attention capture).

Wang and Theeuwes (2020) concluded that feature suppression works well in displays with a few items in which the target is non-salient. Indeed, if the target is non-salient, parallel search is inefficient, and therefore search needs to be serial. Consistent with feature inhibition (Treisman & Sato, 1990) and guided search (Wolfe, Cave, & Franzel, 1989) when search is serial, there can be top-down guidance directing attention to items that are relevant for the task, i.e., direct attention only to items that have the same color as the target (Egeth et al., 1984; Kaptein et al., 1995). Critically, however, Wang and Theeuwes (2020) argued that the signal suppression hypothesis does not apply to conditions in which the target is salient. As such one has to conclude that the signal suppression account maybe less relevant for the attentional capture debate.

## Serial search and capture

The debate about attention capture started in the early 1990s with studies of Theeuwes (1991, 1992) and of Folk et al (1992). The additional singleton paradigm (Theeuwes, 1991, 1992) has been instrumental in this discussion. In this task, observers search for a shape singleton (e.g., a diamond shape between circles) while an irrelevant color singleton was present. The results showed that the irrelevant color singleton captured attention, even though it was never relevant for the search goal. Theeuwes (1992) concluded “*the present study demonstrates that the parallel stage cannot selectively guide the attentive stage to just the known-to-be-relevant target feature*” (p. 605). To be able to conclude this, critically, Theeuwes (1991, 1992) demonstrated that search for the target was indeed performed in parallel across the visual field as set size (5, 7 or 9 elements) did not affected search time (i.e., flat search functions).

**Figure 2:**
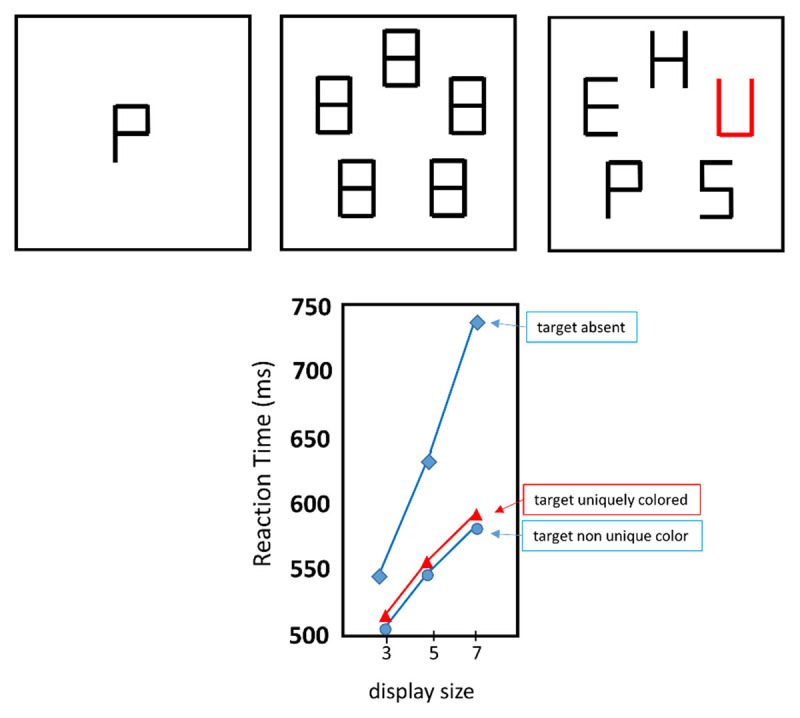
Paradigm and data from Jonides and Yantis (1988). In the first display a target letter was displayed for 1000 ms (in this case the letter P) followed by a premask display for 1000 ms. In the search display one letter had a unique color (in this case red). At chance level this letter could be the target. The results show that observers did not start searching at the red letter even though it was highly salient (search functions for target uniquely colored and target not unique are identical).

It is important to note that if search is not performed in parallel, but instead in a serial fashion, there was never a debate of whether observers are able to ignore salient distractors. The classic work of Jonides and Yantis (1988) provides a good demonstration (see Figure 2). Observers had to search for a target letter among other nontarget letters (set size 3, 5, or 7). All letters were green, except one (which was red). In other words, there was always a salient color singleton present in the display and the question addressed was whether attention was captured by the singleton. With *N* as the number of elements in the display, the singleton happened to be the target on 1/*N* of the trials, indicating that the chance that the salient singleton was the target was the same as for any other letter. Since the salient element was the target at chance level, there was no top-down goal to start searching at the salient singleton (see Figure 2). However, if attention would have been captured by the color singleton and that element happened to be the target (which was the case at chance level: 1/N), observers should be very fast in reporting it. The results showed that observers simply ignored the salient singleton. When the singleton happened to be the target (e.g., an element with a unique color), the search slopes were the same as in the condition in which a non-unique element was the target. Jonides and Yantis (1988) concluded that salient static singletons are treated the same as any other of the non-salient elements in display. Uniqueness in color or luminance were considered not to be sufficient to capture attention when these features were irrelevant to the search goal. However, in this same paradigm, elements appearing with an abrupt onset did have a special status in capturing attention irrespectively of the top–down goal. Theeuwes (2010) argued that static singletons in the Yantis type of search tasks did not capture attention because participants had to search serially for a particular target letter among other nontarget letters. Because search for a target letter among other letters can only be done in a strictly serial way, attention was focused and therefore there was no capture by the salient singleton. Belopolsky, Zwaan, Theeuwes and Kramer (2007) provided evidence for this claim. They used the very same displays as Jonides and Yantis (1988) and manipulated search strategy. Participants either had to divide attention across the display or had to focus spatial attention to a location on the display. When attention was divided across the display, the irrelevant singleton captured attention. However, when attention was highly focused there was no evidence of capture by the salient singleton.

**Figure 3:**
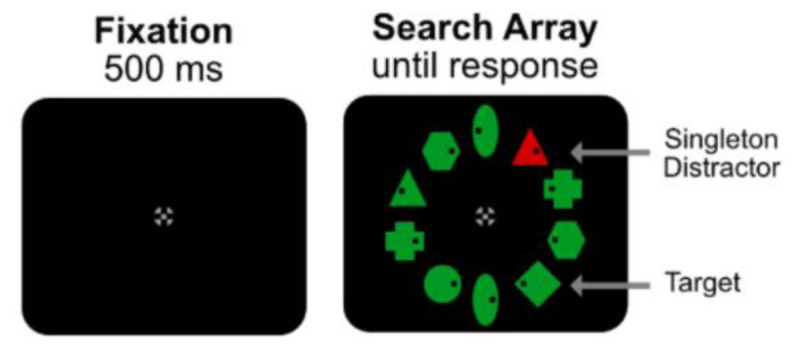
Search displays of Stilwell & Gaspelin (2021). As in Jonides and Yantis (1988), the red singleton was ignored (mean search times for distractor present trials was 926 ms and distractor absent trials was 936 ms).

A recent study by Stilwell and Gaspelin (2021) basically showed the same effect as Jonides and Yantis (1988). In Stilwell and Gaspelin search displays, observers had to find a non-salient target (a diamond) among many other oddly shaped elements (see Figure 3). In some conditions, an irrelevant, but highly salient, distractor was present in the display. The results showed that this highly salient distractor did not capture attention. In fact, instead of showing increased search times for when the distractor was present, the reverse was shown as observers were slightly faster (10 ms) when the distractor was present. Also, the additional probe task showed that there was sub-baseline suppression for the color singleton distractor as observers were less likely to report probe letter presented inside the color singleton relative to the letters placed in the non-singleton elements.

The important message of this study is that observers can ignore a very salient distractor, a conclusion consistent with the results of Jonides and Yantis (1988). The faster search times when the singleton distractor was present relative to when it was absent and the sub-baseline suppression of the distractor singleton location are expected because observers know that the target is green and not red, so they simply ignore locations having red items. This means there is one fewer item to inspect and because (as we assume) search is serial, this will result in the small search time benefit of 10 ms. Reporting fewer probes when presented within the distractor singleton is also to be expected as observers know the target is green and never red. Not attending a color because one knows it is never the target is nothing else than *guided search* (Wolfe, 2021) whereby attention is focused on features (i.e., colors) that are relevant resulting in subset selective search (Egeth et al. 1984; Kaptein et al, 1995). According to this reasoning, there is no need for claiming attentional suppression of the singleton distractor; instead, participants simply search among those items that have the relevant (target) color and ignore everything else (see also Kerzel & Burra, 2020; Kerzel et al., 2021 for a similar reasoning).

As discussed earlier, studies that have shown signal suppression of salient distractors all have either relatively small set sizes and/or heterogeneous displays or both. Heterogeneous displays are used to force observers to search for a specific target feature (the feature search mode); for example, when searching for a diamond target among squares, hexagons, triangles, and pentagons. Because observers have to search for a specific shape among other shapes, they cannot rely on the so called singleton detection mode (Bacon & Egeth, 1994) anymore which is a condition in which the target (e.g., a diamond among circles only) pops-out from the background. Using a small set sizes is typically done to make the experiment more efficient as there are fewer conditions that need to be run.

Even though these typical design features may seem trivial, they have important implications for how search is conducted. Previous research has shown that the saliency of items depends on two factors: local feature contrast (Nothdurft, 1993) and distractor-distractor similarity (Duncan & Humphreys, 1989). Local feature contrast refers to how different an item is from nearby items (Nothdurft, 1993). In a display with only a few elements equally spaced around the fixation point, the elements are relatively far apart, reducing the local feature contrast. In addition, heterogeneous features (e.g., squares, hexagons, and circles) used to force a feature search mode negatively affect the distractor-distractor similarity. It was shown that distractor heterogeneity reduces search efficiency, resulting in serial search (Duncan & Humphreys, 1989). As such, one could argue that the “feature search mode” is not a top-down attentional set but instead is induced by displays that require discrimination of individuated items resulting in (partly) serial search for the non-salient target (Theeuwes, 2010).

What this basically means is that in all studies showing signal suppression, the target is not salient enough to rely on parallel search to find it. Instead, observers use (clump-wise) serial search because the target is relatively difficult to find. Because observers use clump-wise serial search they simply ignore salient singleton as was already demonstrated by the classic study of Jonides and Yantis in 1988. The notion of clump-wise search was first proposed by Pashler (1987) to account for inconsistencies in search slopes of conjunction search tasks. According to this notion, there is serial search over larger clumps of items, and a parallel search within these clumps.

**Figure 4:**
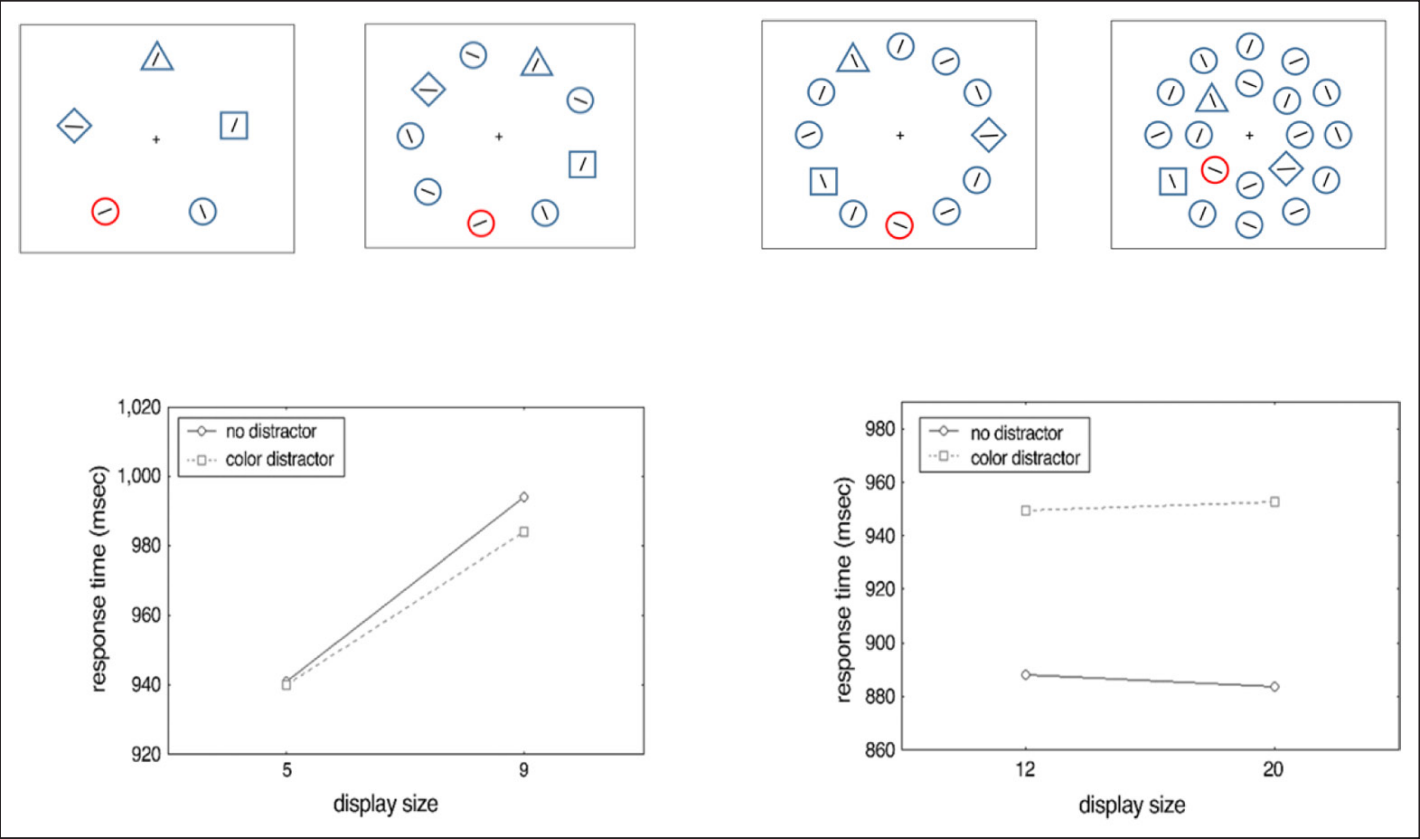
Paradigm and data from Theeuwes (2004). Observers search for a specific target feature (so-called feature search mode). At the top left: search for a diamond in displays consisting of either 5 or 9 nontarget elements; at the top right: search for a diamond in displays consisting of either 12 or 20 nontarget elements. At the bottom left: in small displays this leads to serial search and no interference of the color distractor; at the bottom right: it leads to parallel search (flat search functions) and a large inference of the color distractor (attentional capture of about 80 ms).

One could argue that our reasoning is problematic. Signal suppression only occurs when observers engage in the feature search mode, and because heterogeneous displays are needed to induce the feature search mode, observers rely on (partly) serial search as the target is difficult to find. This would imply that it would be impossible to test whether observers can search for a specific feature (i.e., feature search mode) and *at the same time* employ efficient, parallel search across the visual field. While this is usually the case, it is possible to create conditions in which participants need to search for a *specific* feature (feature search mode) while at the same time search is conducted in parallel across the visual field. For example, Theeuwes (2004) used displays in which distractor shapes were heterogeneous (e.g., squares, diamonds, triangle and circles), which required participants to search for a specific feature (inducing the feature search mode; see Figure 4). Yet, instead of having only a few items in the display, Theeuwes (2004) used up to 20 items and showed flat search functions signifying parallel search. Even though the feature search mode had to be used, there was no evidence to any top-down suppression as the irrelevant singleton captured attention. Theeuwes (2004) also showed that when small set sizes were used, search was serial and there was no longer evidence of attentional capture. However, for the large set sizes, when search was conducted in parallel there was evidence of attentional capture (see also Wang & Theeuwes, 2020). It is important to realize that there was a large capture effect even though observers must have used the assumed “top-down” feature search mode (see Figure 4).

One important question is why observers use clump-wise search in some displays (on the left in Figure 4) and parallel search in others (on the right in Figure 4). Theeuwes (1994) suggested the notion of a modifiable attentional window (for similar ideas see Humphreys & Müller, 1993). Theeuwes (1994) argued that within the attentional window, processing occurs in parallel. In order to keep an acceptable signal-to-noise-ratio, the size of the window is adjusted to the discriminability of the target. In the example above for the small set sizes, both the local feature contrast (Nothdurft, 1993) and distractor-distractor similarity (Duncan & Humphreys, 1989) are relatively low making it harder to discriminate the target among the nontargets. Therefore, to maintain an acceptable signal-to-noise-ratio observers employed a smaller attentional window resulting in clump-wise serial search with search slopes of about 12 ms/item. For the large set sizes, there is enough local feature contrast (i.e., elements are positioned relatively close together) and the distractor-distractor similarity is relatively high as there are mostly circles as non-targets. This gives parallel search with flat search functions of about 0 ms/item, but large capture effect of about 65 msec. Even though in his original conception of the attentional window, Theeuwes (1994) argued that the size of the attentional window could be strategically adopted to the task demands (see also Belopolsky et al., 2007), at this point it is unclear whether the size of the attentional window is under top-down control. It is feasible that the strategy is induced by the search display without much, if any, top-down control.

## Boundary conditions

We argue that if finding the target requires a fine discrimination of each individuated item’s feature, search cannot proceed in parallel; instead search needs to proceed serially in clumps adopting a small attentional window. In cases in which the attentional window is small, there will be no capture even when the distractor is very salient. As shown by Jonides and Yantis (1988), a salient element is not prioritized in search as observers simply covertly select a random location to initiate search. If observers know that the target has a particular color, then serial search may start at those locations containing the target color and not the color of the distractor (Egeth et al 1984; Kaptein et al., 1995). This explains why in many studies testing the signal suppression hypothesis, observers are often faster when a distractor is present then when it is absent. For example, in a recent study, Chang and Egeth (2019) found that observers were significantly faster and more accurate on distractor present trials than on absent trials. Chang and Egeth were surprised by this finding and argued “*the actual reversal of the capture effect is a striking finding*” (p. 1731). The same capture reversal was reported by Gaspelin, Leonard and Luck (2015) and Stilwell and Gaspelin (2021). Even though these authors may find these effects striking, it is not that surprising if search is conduced serially (as we claim here) as observers simply have one less item to search when the distractor is present than when it is absent (see also Ruthruff et al., 2021 for a similar conclusion).

Our analysis has many parallels to that of Liesefeld, Liesefeld and Muller, (2021; Liesefeld & Muller, 2020) who claim that “*the remaining discrepancies in the attentional-capture debate can be resolved by a simple assumption: observers do not use the priority map when this map is useless to solve the task*” (Liesefeld et al., 2021; p.600). Liesefeld et al. (2021) argue that the priority map is only useful if it reliably guides attention to the target. If the target is not salient enough search needs to be conducted in “*clumps of adjacent objects in parallel in a spatially systematic fashion*” (Liesefeld & Muller, 2020; p. 233). Liesefeld et al. point out that in the tasks used by Gaspelin and Luck, using the priority map is likely inefficient, making observers resort to clump-scanning mode. They agree with the findings of Wang and Theeuwes (2020) that in sparse heterogeneous displays, targets are non-salient such that search proceeds clump-wise and serial. While we fully agree with Liesefeld and colleagues that in these type of displays, search proceeds in a clump-wise fashion, we do not adhere the position that during clump-wise search, the priority map plays no role anymore. On the contrary, we assume that during serial clump-wise search, there is massive guidance by the priority map (as assumed by Guided Search, Wolfe, 1994 and Feature Suppression; Treisman & Sato, 1990). Indeed, search is guided by the display characteristics and even within a search episode during a trial, the attentional window can vary in size. For example, groups of homogenous nontargets elements can be discarded quickly as a large clump of items. If the display is more noisy, (for example, in non-homogenous displays typically used to induce the feature search mode; see Figure 1 bottom) the size of the window is reduced to maintain an acceptable signal-to-noise ratio. Clumps maybe small possibly even encompassing only one item. As such we do not adhere the position that there is a dichotomy of search strategies (as assumed by Liesefeld and colleagues) but instead we assume that the search strategy employed depends on the discriminability of the target and display characteristics (e.g., spatially adjacent clumps of homogenous non-target elements that can be discarded quickly in one go).

## Final remarks

It should be noted that display size itself nor the salience of the distractor are the determining factors whether capture can be avoided. Indeed, several recent studies have shown that capture by a very salient distractor can be avoided in displays with 10 to 30 elements (Chang, Niebur, & Egeth, 2021; Stilwell and Gaspelin, 2021; Stilwell, Egeth, & Gaspelin, 2022). Yet, in all these studies the target was not salient suggesting that the preattentive processing was omitted and search proceeded in clumps across the visual field. In those circumstances, even the most salient distractor will be avoided. Whether or not top-down control is gained through signal suppression of the salient distractor (as in Gaspelin et al., (2015), inhibition of features that are unlike the target (as in feature inhibition; Treisman & Sato, 1990) or whether attention is enhanced for features that are relevant (as in Guided Search of Wolfe, 1990) is still a matter of debate. Recently, Chang and Egeth (2019) argued that both processes may play a role as they showed that both enhancement and suppression flexibly guided attention.

Along these lines, a recent study by Lien, Ruthruff, and Hauck (2021) suggested that signal suppression of salient distractors assumed by Gaspelin is less likely to be the mechanism by which top-down control is gained. Lien et al. (2021) replicated Gaspelin et al.’s probe suppression effect when searching for a green diamond while ignoring a single red distractor. Critically however, instead of one salient red distractor, they presented three red distractors and showed below baseline suppression for all distractors that were red. In this condition, the display consisted of three green items (one of which was the diamond target) and three red distractor items. Ruthruff et al. (2021) argued that for signal suppression to occur, one does not need a salient singleton and an “attend to me signal” (Gaspelin et al., 2015) but instead observers can direct attention to relevant colors only (Guided Search; Wolfe, 1990). It is important to note that the data of Lien et al. (2011) suggest that search was serial even though Lien et al. did not interpret their findings as such. Indeed, again as others have shown “the striking reversal of the capture effect” Lien et al (2011) showed that distractor absent trials gave the slowest search times as all 6 elements needed to be inspected (resulting in a mean RT of 688 ms). When one red distractor was present, participants were faster as one less item needed to be searched (a mean RT of 677 ms). Critically, when there were 3 red distractors and 3 green (relevant) items, search times were even faster (a mean RT of 641 ms) as in this condition only 3 relevant items needed to be inspected. Along similar lines, because participants direct attention to the green items only, it is not surprising that participants are less likely to report probes presented at locations of the (ignored) red items. In this sense, it is debatable whether not reporting probes presented at locations of items that are not searched should be considered to as suppression. Items with irrelevant colors are simply ignored and as such the probes presented at these locations are simply not reported. In this sense, the Ruthruff et al. (2021) findings are the same as those conducted in the 80s and 90s in which it was shown that observers not search all elements in a display but only those that have the same color as that of the target (Kaptein et al., 1995; Egeth et al 1984) an effect referred to as “sub-set selective search”.

A recent study by Ma and Abrams (2022) showed a similar effect. In this study, participants had to identify the most prevalent shape (either circles or squares) in an array of 6 elements. These 6 elements all had the same color (all red or all green) or contained one color singleton distractor with an unknown color (e.g., 5 elements were green and one was gray). The results showed that when the color singleton distractor was present, participants were faster than when it was absent, again suggesting what was labelled before as “a striking reversal of the capture effect” (c.f., Chang and Egeth, 2019). Moreover, Ma and Abrams showed that when a probe letter was presented at the location of the color singleton distractor, participants were less likely to report that letter. The authors interpreted these findings as evidence for “proactive suppression” of an unknown color singleton distractor. According to our interpretation, there is no suppression and there is no striking reversal of the capture effect. To determine the most prevalent shape, participants search serially among the relevant subset consisting of either 6 elements (when the color singleton distractor is absent) or 5 elements (when the color singleton distractor is present). Serial search among 5 elements is faster than among 6 elements explaining the “striking capture reversal effect”. Because participants search among the relevant set only (Kaptein et al., 1995; Egeth et al 1984), they simply ignore the color singleton. It is not surprising that probe letters at the location of the ignored color singleton are reported much often than letter presented at the location of the relevant set. As such, there is no need to infer anything like “suppression”; participants perform subset selective search among the relevant set only and ignore those elements that are irrelevant (Kaptein et al, 1995).

Even though we adhere the position that when search proceeds in parallel across the display, selection is bottom-up with no possibility to prevent attentional capture and when search is (partly) serial in clumps, attentional capture can be prevented, it is important to note that search slopes themselves are not an adequate diagnostic to infer whether search was parallel or serial. Indeed, there are many factors that influence search slopes above and beyond the strict parallel-serial dichotomy (see Liesefeld & Müller, 2020; Palmer, 1995; Townsend, 1971). For example, when search proceeds in clumps of items, the size of the window may vary within and across trials depending on the exact display characteristics encountered during a particular trial. This may give search slopes that are rather flat while search was conducted serially in relatively large clumps. It should be noted that the steepness of a search slope depends very much on the speed with which a participant can decide that the item that just was selected during search is *not* the target. If the item selected resembles the target, it is difficult to disengage attention from the item and proceed to the next item which may give rise to relative steep slopes. If however the item selected is very much unlike the target it can be discarded quickly resulting is relatively flat search functions (see Born, Kerzel & Theeuwes, 2011).

## Conclusions

It is evident that observers can avoid salient distractors, possibly by a mechanism that can be labeled as *signal suppression* (Gaspelin & Luck, 2015), *feature inhibition* (Treisman & Sato, 1990), *guided search* (Wolfe et al, 1989) or *subset selective search* (Egeth et al., 1984; Lien at al., 2021; Kaptein et al., 1995). Yet, this only occurs in conditions in which the target is not easy to find (non-salient), and observers use a search strategy involving serial clump-wise search. As argued (Theeuwes, 1994; Belopolsky, et al., 2007), serial search involves a small attentional window (focused attention or zoom-lens/spotlight) and salient signals outside the focus of attention that have no effect on search. In these circumstances, search is very much under top-down control guided by knowledge regarding the target (guided search) and by knowledge about what is not the target (signal suppression, feature inhibition).

If, however, the target is salient and observers use parallel search to find the target, any salient distractor will capture attention. The price for searching in parallel is that any salient signal within the (parallel) attentional window (Theeuwes, 1994) can capture attention and disrupt the search for the target. After attention is captured by an irrelevant singleton, top-down knowledge may allow for a very fast disengagement of attention (Theeuwes, Atchley, & Kramer, 2000; Theeuwes, 2010).
